# Factors associated with posttraumatic meningitis among traumatic head injury patients: a nationwide study in Japan

**DOI:** 10.1007/s00068-019-01224-z

**Published:** 2019-09-03

**Authors:** Yusuke Katayama, Tetsuhisa Kitamura, Kosuke Kiyohara, Junya Sado, Tomoya Hirose, Tasuku Matsuyama, Takeyuki Kiguchi, Jotaro Tachino, Shunichiro Nakao, Yutaka Umemura, Yuko Nakagawa, Takeshi Shimazu

**Affiliations:** 1 Department of Traumatology and Acute Critical Medicine, Osaka University Graduate School of Medicine, 2-15, Yamada-oka, Suita, Japan; 2 Division of Environmental Medicine and Population Sciences, Department of Social and Environmental Medicine, Osaka University Graduate School of Medicine, 2-15, Yamada-oka, Suita, Japan; 3 Department of Food Science, Faculty of Home Economics, Otsuma Women’s University Tokyo, 12, Sanban-cho, Chiyoda-ku, Tokyo, Japan; 4 Emergency and Critical Care Center, Osaka Police Hospital, 10-31, Kitayama-cho, Tennoji-ku, Osaka, Japan; 5 Department of Emergency Medicine, Kyoto Prefectural University of Medicine, 465 Kajiicho, Hiroko-ji noboru, Kawaramachi-dori, Kamigyo-ku, Kyoto, Japan; 6 Kyoto University Health Services, Yoshida-honmachi, Sakyo-ku, Kyoto, Japan; 7 Department of Emergency and Critical Care, Osaka General Medical Center, 3-1-56, Bandai-Higashi, Sumiyoshi-ku, Osaka, Japan

**Keywords:** Posttraumatic meningitis, Decompressive craniectomy, Cerebrospinal fluid leakage, Burr hole surgery in emergency department

## Abstract

**Purpose:**

Posttraumatic meningitis is one of the severe complications that can result in increased mortality and longer hospital stay among trauma patients. Factors such as cerebrospinal fluid (CSF) fistula and basilar skull fracture are associated with posttraumatic meningitis. However, it remains unclear whether procedures such as burr hole surgery in the emergency department and decompressive craniectomy are associated with posttraumatic meningitis. The aim of this study was to assess factors associated with posttraumatic meningitis with a nationwide hospital-based trauma registry in Japan.

**Methods:**

This was a retrospective observational study with a 12-year study period from January 2004 to December 2015. We included trauma patients registered in the Japanese Trauma Data Bank, whose head Abbreviated Injury Scale score was ≥ 3 in this study. The main endpoint was the occurrence of meningitis during hospitalization. Multivariable logistic regression analysis was used to assess independent parameters associated with posttraumatic meningitis such as CSF fistula, burr hole surgery in the emergency department, and decompressive craniectomy.

**Results:**

Among 60,390 head injury patients with head AIS score 3 or more, 284 (0.5%) patients had posttraumatic meningitis. Factors associated with posttraumatic meningitis were burr hole surgery in the emergency department (adjusted odds ratio [AOR] 2.158 [95% confidence interval (CI) 1.401–3.325]), decompressive craniectomy (AOR 2.123 [95% CI 1.506–2.993]), external ventricular drainage (AOR 1.843 [95% CI, 1.157–2.935]), CSF leakage (AOR 3.328 [95% CI 2.205–5.022]), and basilar skull fracture (AOR 1.651 [95% CI 1.178–2.314]).

**Conclusions:**

In this population of trauma patients, burr hole surgery in the emergency department and decompressive craniectomy was associated with posttraumatic meningitis.

## Introduction

Head injury occurs in about 25% of trauma patients in Japan [1], and mortality remains high. In addition, many head injury survivors have a poor neurological outcome due to diffuse axonal injury [2]. Meningitis can be a severe complication with a negative influence on the outcome after craniocerebral trauma. There are reports about high mortality rates from 29% to 57.9% due to this infection [3–5]. Previous studies revealed that basilar skull fracture and cerebrospinal fluid (CSF) leakage were also associated with posttraumatic meningitis [6, 7]. In addition, the length of time of external ventricular drainage, emergency operation, and operation time over 4.5 h was also reported to relate to the occurrence of postoperative meningitis in patients undergoing a neurosurgical operation [8, 9].

If it takes time to transport severe trauma patients from areas, where there are no neurosurgeons to distant medical institutions, their prognosis would become worse [10]. In addition, it may take time to prepare a hospital operating room when an emergency operation is required for severe head injury patients. Thus, burr hole surgery is sometimes performed in the emergency department for some patients, and decompressive craniectomy for patients with traumatic brain injury and refractory intracranial hypertension has resulted in a favorable neurological outcome [11–15]. However, it is unclear whether burr hole surgery performed in the emergency department, the procedures performed during the neurosurgical operation and repeat surgery are associated with posttraumatic meningitis in patients with traumatic head injury.

The Japanese Trauma Data Bank (JTDB) is a nationwide trauma registry in Japan that is managed by The Japanese Association for The Surgery of Trauma. Data registration in the JTDB was launched in 2003, and approximately 230,000 emergency trauma patients were enrolled by 2015 [1]. With the use of data from the JTDB registry, the aim of this study was to evaluate the association between the occurrence of posttraumatic meningitis and factors such as patient characteristics, type of surgery, and procedures performed during the surgery among the traumatic head injury patients with head AIS score 3 or more.

## Methods

### Study design, population, and setting

This was a retrospective observational study that used data of emergency trauma patients registered in the JTDB. The study period was the 12-year period from January 2004 to December 2015. In this study, we included patients with a head Abbreviated Injury Scale (AIS) score of 3 or more who were transported to a JTDB-participating hospital and were registered in the JTDB registry [16]. We excluded those patients who were in cardiopulmonary arrest on hospital arrival, whose mandatory data were missing. Cardiopulmonary arrest on hospital arrival was defined as a patient, whose systolic blood pressure was 0 mmHg and/or heart rate was 0 bpm on hospital arrival. From the JTDB database, we extracted factors such as age, sex, past medical history, type of trauma, CSF leakage, type of skull fracture, burr hole surgery in the emergency department, operative procedure, and procedures performed during the neurosurgery, and repeat surgery within 48 h of the first surgery. In this study, we defined multiple trauma as head trauma patients with AIS score of 3 or more in body areas other than the head. In addition, we defined CSF leakage as a patient for whom AIS code 150204.3 was recorded. In addition, those with AIS codes 150408.4, 150406.4, 150404.3, 150402.2, and 150400.2 were defined as having a skull fracture, and those with AIS codes 150206.4, 150204.3, 150202.3, and 150200.3 were defined as having a basilar skull fracture. The primary operative method of neurosurgery was classified as burr hole surgery or craniotomy, and the procedures performed during the neurosurgery were classified as evacuation of hematoma, decompressive craniectomy, lobectomy, duraplasty, cranioplasty, and external ventricular drainage. This study was approved by the ethics committee of Osaka University Graduate School of Medicine (No. 16260). Personnel identifiers were removed beforehand from the JTDB database, and thus, the patients’ right to informed consent was waived.

### Japanese Trauma Data Bank

The JTDB was launched in 2003 by the Japanese Association for the Surgery of Trauma (Trauma Surgery Committee) and the Japanese Association for Acute Medicine (Committee for Clinical Care Evaluation) [1, 17], similar to trauma databases in North America, Europe, and Oceania [18]. By 2016, 256 major emergency medical institutions across Japan had been registered in the JTDB database [1]. These hospitals have equal ability to that of Level I trauma centers in the United States. Data were collected via the Internet from participating institutions. The physicians and medical assistants who attended the AIS-coding course were the main registers of the data [19].

The JTDB captures trauma patient data on age, sex, mechanism of injury, AIS code (version 1998), Injury Severity Score (ISS), vital signs on hospital arrival, date and time series from hospital arrival to discharge, medical treatments such as interventional radiology, surgical operation, and CT scanning, complications, and mortality at discharge [19]. ISS was calculated from the top three scores of AIS in nine sites classified by AIS codes.

### Endpoint

The endpoint was the occurrence of meningitis during hospitalization, and we defined this meningitis as posttraumatic meningitis. We extracted data on the occurrence of meningitis from the JTDB registry.

### Statistical analysis

In this study, we assessed factors associated with the occurrence of posttraumatic meningitis with multivariable logistic regression analysis and calculated the adjusted odds ratio (AOR) and 95% confidential interval (CI). Multivariable logistic regression analysis was performed by forced enter-method. The independent parameters were age group (0–9 years, 10–19 years, 20–29 years, 30–39 years, 40–49 years, 50–59 years, 60–69 years, 70–79 years, 80 years, or older), sex, diabetes, implementation of hemodialysis, multiple trauma or single head trauma, presence or absence of CSF leakage, type of skull fracture, implementation of burr hole surgery in the emergency department, first neurosurgical operation, procedures performed during the first neurosurgical operation, and repeat surgery within 48 h of the first surgery. These independent parameters that were biologically essential and considered to be associated with outcomes were included in the multivariable regression analysis-based previous studies [3–9, 20, 21]. As a further sub-analysis, we divided these patients into the single head trauma group and the multiple trauma group [22], and these independent parameters associated with posttraumatic meningitis were also assessed with multivariable logistic regression analysis. All tests were two-tailed, and a *P* value of < 0.05 was considered statistically significant. Statistical analysis was performed by SPSS version 23.0J (IBM Crop., Armonk, NY, USA). This manuscript was written based on the STROBE statement to assess the reporting of cohort and cross-sectional studies.

## Results

**Fig. 1:**
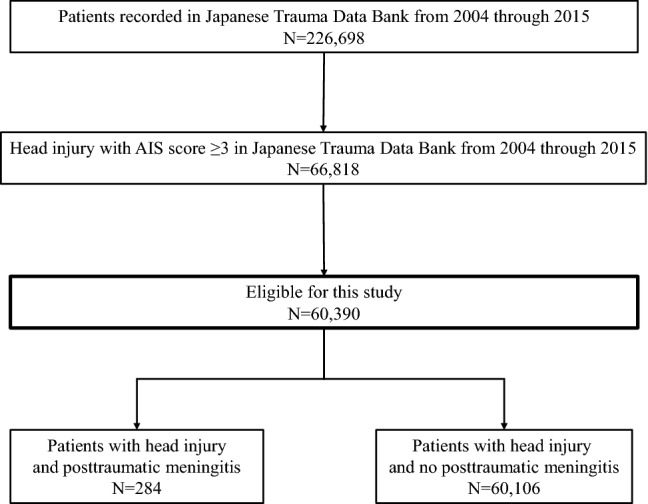
Patient flow in this study

Figure 1 shows the patient flow in this study. In total, 226,698 emergency patients were registered in the JTDB from 2004 to 2015, and 66,818 patients had head trauma with a head AIS score of 3 or more. Among these head trauma patients, 4901 patients were in cardiopulmonary arrest on hospital arrival, and 1527 patients who did not have the necessary data (missing age: 65 patients, missing sex: 16 patients, and missing outcome data: 1446 patients) were excluded from this study, leaving 60,390 patients suitable for analysis. Among these 60,390 patients, 284 (0.5%) had posttraumatic meningitis.

Table 1 shows the patient characteristics in this study. The median age was 63 (interquartile range [IQR] 38–76) years, 68.4% were males, and the median Glasgow Coma Scale score was 13 (IQR 8–15). In total, 14,085 patients (23.3%) had skull fractures and 8388 patients (13.9%) had basilar skull fractures. There were 2033 patients (3.4%) with CSF leakage, and 1468 patients (2.4%) had undergone burr hole surgery in the emergency department. As the first neurosurgical operation, craniotomy was performed in 7128 patients (11.5%), and burr hole surgery was performed in 2910 (4.5%). The procedures performed during the first neurosurgical operation included evacuation of hematoma in 8262 patients (13.7%), decompressive craniectomy in 2495 (4.1%), lobectomy in 416 (0.7%), duraplasty in 267 (0.4%), cranioplasty in 437 (0.7%), and external ventricular drainage in 786 patients (1.3%). Among those patients who underwent repeat surgery within 48 h of the first surgery, 928 (1.5%) received a craniotomy and 235 (0.4%) burr hole surgery.

**Table 1:** Demographic and clinical characteristics of head injury patients with head AIS ≥ 3

Characteristic	Total (*n* = 60,390)
Age, years, median (IQR)	63 (38–76)
0–9	2574 (4.3)
10–19	4306 (7.1)
20–29	4695 (7.8)
30–39	4196 (6.9)
40–49	4818 (8.0)
50–59	6285 (10.4)
60–69	10,394 (17.2)
70–79	12,181 (20.1)
Over 80	10,941 (18.1)
Male, *n* (%)	41,334 (68.4)
Glasgow Coma Scale, median (IQR)	13 (8–15)
Past medical history, *n* (%)	
Diabetes mellitus	5672 (9.4)
Dialysis	861 (1.4)
Type of trauma, *n* (%)	
Single TBI	50,975 (84.4)
Multiple trauma	9415 (15.6)
Type of skull fracture, *n* (%)	
Skull fracture	14,085 (23.3)
Basilar skull fracture	8388 (13.9)
Cerebrospinal fluid leakage, *n* (%)	2033 (3.4)
Burr hole surgery in the emergency department, *n* (%)	1468 (2.4)
First neurosurgical operation, *n* (%)	
Craniotomy	7128 (11.8)
Burr hole surgery	2910 (4.8)
Procedures performed during the first neurosurgical operation, *n* (%)	
Evacuation of hematoma	8262 (13.7)
Decompressive craniectomy	2495 (4.1)
Lobectomy	416 (0.7)
Duraplasty	267 (0.4)
Cranioplasty	437 (0.7)
External ventricular drainage	786 (1.3)
Repeat surgical procedure performed within 48 h of first surgery, *n* (%)	
Craniotomy	928 (1.5)
Burr hole surgery	235 (0.4)

*AIS* Abbreviated Injury Scale, *IQR* interquartile range, *TBI* traumatic brain injury

Table 2 shows the results of the association between the occurrence of posttraumatic meningitis and various factors. Male sex (AOR 1.472 [95% CI 1.102–1.965]), multiple trauma (AOR 1.415 [95% CI 1.054–1.900]), basilar skull fracture (AOR 1.651 [95% CI 1.178–2.314]), CSF leakage (AOR 3.328 [95% CI 2.205–5.022]), burr hole surgery in the emergency department (AOR 2.158 [95% CI 1.401–3.325]), craniotomy (AOR 4.629 [95% CI 3.087–6.942]), and burr hole surgery as an operative method (AOR 2.259 [95% CI 1.449–3.523]) were associated with the occurrence of posttraumatic meningitis. Regarding the procedure performed during the first neurosurgical operation, decompressive craniectomy (AOR 2.123 [95% CI 1.506–2.993]) and external ventricular drainage (AOR 1.843 [95% CI 1.157–2.935]) were associated with posttraumatic meningitis. For repeat surgery within 48 h of the first surgery, both craniotomy (AOR; 3.193 [95% CI 2.137–4.769]) and burr hole surgery (AOR; 4.222 [95% CI 2.194–8.124]) were associated with posttraumatic meningitis.

**Table 2:** Factors associated with posttraumatic meningitis

	Meningitis % (*n*/*N*)	Adjusted OR (95% CI)		*P* value
Age group (years)				
0–9	0.5 (12/2574)	0.923 (0.461–1.847)		0.821
10–19	0.5 (20/4306)	0.718 (0.398–1.292)		0.269
20–29	0.8 (37/4695)	1.239 (0.750–2.049)		0.403
30–39	0.7 (29/4196)	Reference		
40–49	0.5 (26/4818)	0.849 (0.493–1.464)		0.557
50–59	0.6 (38/6285)	1.041 (0.631–1.716)		0.876
60–69	0.5 (47/10,394)	0.765 (0.473–1.239)		0.277
70–79	0.4 (46/12,181)	0.797 (0.489–1.297)		0.360
Over 80	0.3 (29/10,941)	0.749 (0.436–1.286)		0.294
Sex				
Male	0.5 (221/41,334)	1.472 (1.102–1.965)		0.009
Female	0.3 (63/19,056)	Reference		
Past medical history				
Diabetes mellitus				
(+)	0.4 (25/5672)	1.134 (0.735–1.750)		0.571
(−)	0.5 (259/54,718)	Reference		
Dialysis				
(+)	0.3 (3/861)	0.925 (0.290–2.959)		0.896
(−)	0.5 (281/59,529)	Reference		
Type of trauma				
Single TBI	0.4 (223/50,975)	Reference		
Multiple trauma	0.6 (61/9415)	1.415 (1.054–1.900)		0.021
Type of skull fracture				
Skull fracture				
(+)	0.8 (108/14,085)	1.163 (0.900–1.504)		0.249
(−)	0.4 (176/46,305)	Reference		
Basilar skull fracture				
(+)	1.2 (99/8388)	1.651 (1.178–2.314)		0.004
(−)	0.4 (185/52,002)	Reference		
CSF leakage				
(+)	2.7 (54/2033)	3.328 (2.205–5.022)		< 0.001
(−)	0.4 (230/58,127)	Reference		
Burr hole surgery in ED				
(+)	3.3 (49/1468)	2.158 (1.401–3.325)		< 0.001
(−)	0.4 (235/58,922)	Reference		
First neurosurgical operation				
Craniotomy				
(+)	1.8 (125/7128)	4.629 (3.087–6.942)		< 0.001
(−)	0.3 (159/53,262)	Reference		
Burr hole surgery				
(+)	1.9 (54/2910)	2.259 (1.449–3.523)		< 0.001
(−)	0.4 (230/57,480)	Reference		
Procedures performed during the first neurosurgical operation				
Evacuation of hematoma				
(+)	1.6 (134/8262)	0.792 (0.528–1.188)		0.260
(−)	0.3 (150/52,128)	Reference		
Decompressive craniectomy				
(+)	3.4 (84/2495)	2.123 (1.506–2.993)		< 0.001
(−)	0.3 (200/57,895)	Reference		
Lobectomy				
(+)	2.9 (12/416)	0.871 (0.460–1.651)		0.673
(−)	0.5 (272/59,974)	Reference		
Duraplasty				
(+)	2.6 (7/267)	0.871 (0.384–1.975)		0.740
(−)	0.5 (477/60,123)	Reference		
Cranioplasty				
(+)	2.1 (9/437)	1.284 (0.619–2.663)		0.502
(−)	0.5 (275/59,953)	Reference		
External ventricular drainage				
(+)	3.6 (28/786)	1.843 (1.157–2.935)		0.010
(−)	0.4 (256/59,604)	Reference		
Repeat surgical procedure performed within 48 h of first surgery				
Craniotomy				
(+)	4.6 (43/928)	3.193 (2.137–4.769)		< 0.001
(−)	0.4 (241/59,462)	Reference		
Burr hole surgery				
(+)	5.1 (12/235)	4.222 (2.194–8.124)		< 0.001
(−)	0.5 (272/60,155)	Reference		

*TBI* traumatic brain injury, *CSF* cerebrospinal fluid, *ED* emergency department, *OR* odds ratio, *CI* confidence interval

Table 3 shows the results of sub-analysis divided into single head trauma and multiple trauma. In the group with single head trauma, burr hole surgery in the emergency department (AOR 2.456 [95% CI 1.522–3.965]), craniotomy (AOR 3.587 [95% CI 2.248–5.723]), and burr hole surgery as an operative method (AOR 2.112 [95% CI 1.277–3.493]) were associated with posttraumatic meningitis. As a procedure performed during the first neurosurgical operation, only decompressive craniectomy (AOR 2.292 [95% CI 1.562–3.363]) was associated with posttraumatic meningitis. In addition, both craniotomy (AOR 3.044 [95% CI 1.952–4.746]) and burr hole surgery (AOR 3.387 [95% CI 1.531–7.492]) were associated with posttraumatic meningitis when repeat surgery was performed within 48 h of the first surgery. In contrast, only craniotomy as a neurosurgical operative method (AOR 9.381 [95% CI 4.199–20.958]) was associated with posttraumatic meningitis in the multiple trauma group. As a procedure performed during the first neurosurgical operation, external ventricular drainage (AOR 2.790 [95% CI 1.113–6.992]) was associated with posttraumatic meningitis, but the evacuation of hematoma was inversely related (AOR 0.417 [95% CI 0.179–0.970]). In addition, both craniotomy (AOR 4.372 [95% CI 1.661–11.503]) and burr hole surgery (AOR 6.368 [95% CI 1.825–22.224]) were associated with posttraumatic meningitis as a repeat surgical procedure performed within 48 h of the first surgery.

**Table 3:** Odds ratios of each variable for posttraumatic meningitis among patients with single TBI and multiple trauma

	Single TBI			Multiple trauma		
	% (*n*/*N*)	Adjusted OR (95% CI)	*P* value	% (*n*/*N*)	Adjusted OR (95% CI)	*P* value
Age group (years)						
0–9	0.3 (8/2325)	0.662 (0.293–1.496)	0.321	1.6 (4/249)	3.188 (0.736–13.805)	0.121
10–19	0.3 (11/3591)	0.461 (0.223–0.953)	0.037	1.3 (9/715)	2.409 (0.711–8.164)	0.158
20–29	0.8 (28/3700)	1.107 (0.633–1.938)	0.721	0.9 (9/995)	2.166 (0.647–7.247)	0.210
30–39	0.8 (25/3319)	Reference		0.5 (4/877)	Reference	
40–49	0.4 (17/3952)	0.632 (0.336–1.190)	0.155	1.0 (9/866)	2.403 (0.715–8.083)	0.156
50–59	0.6 (33/5237)	0.972 (0.567–1.668)	0.918	0.5 (5/1048)	1.291 (0.336–4.959)	0.710
60–69	0.4 (37/8838)	0.653 (0.384–1.110)	0.115	0.6 (10/1556)	1.425 (0.431–4.709)	0.562
70–79	0.4 (40/10,451)	0.749 (0.442–1.270)	0.284	0.3 (6/1730)	0.960 (0.261–3.538)	0.952
over 80	0.3 (24/9562)	0.659 (0.364–1.191)	0.167	0.4 (5/1379)	1.391 (0.358–5.414)	0.634
Sex						
Male	0.5 (173/34,904)	1.425 (1.029–1.974)	0.033	0.7 (48/6430)	1.533 (0.812–2.891)	0.187
Female	0.3 (50/16,071)	Reference		0.4 (13/2985)	Reference	
Past medical history						
Diabetes mellitus						
(+)	0.5 (23/5010)	1.225 (0.775–1.935)	0.386	0.3 (2/662)	0.659 (0.155–2.815)	0.574
(−)	0.4 (200/45,965)	Reference		0.7 (59/8753)	Reference	
Dialysis						
(+)	0.3 (2/793)	0.666 (0.161–2.747)	0.574	1.5 (1/68)	3.771 (0.468–30.402)	0.213
(−)	0.4 (221/50,182)	Reference		0.6 (60/9347)	Reference	
Type of fracture						
Skull fracture						
(+)	0.7 (90/12,102)	1.218 (0.913–1.624)	0.179	0.9 (18/1983)	1.023 (0.564–1.856)	0.941
(−)	0.3 (133/38,873)	Reference		0.6 (43/7432)	Reference	
Skull base fracture						
(+)	1.2 (81/6853)	1.830 (1.251–2.676)	0.002	1.2 (18/1535)	1.253 (0.595–2.640)	0.553
(−)	0.3 (142/44,122)	Reference		0.5 (43/7880)	Reference	
CSF leakage						
(+)	2.8 (45/1623)	3.543 (2.244–5.723)	< 0.001	2.2 (9/410)	2.334 (0.874–6.235)	0.091
(−)	0.4 (178/49,352)	Reference		0.6 (52/9005)	Reference	
Burr hole surgery at ED						
(+)	3.5 (39/1118)	2.456 (1.522–3.965)	< 0.001	2.9 (10/350)	1.620 (0.611–4.291)	0.332
(−)	0.4 (184/49,857)	Reference		0.6 51/9065	Reference	
First neurosurgical operation						
Craniotomy						
(+)	1.6 (101/6271)	3.587 (2.248–5.723)	< 0.001	2.8 (24/857)	9.381 (4.199–20.958)	< 0.001
(−)	0.3 (110/43,692)	Reference		0.4 (37/8558)	Reference	
Burr hole surgery						
(+)	1.7 (43/2471)	2.112 (1.277–3.493)	0.004	2.5 (11/439)	2.278 (0.873–5.944)	0.092
(−)	0.4 (180/48,504)	Reference		0.6 (50/8976)	Reference	
Procedures performed during the first neurosurgical operation						
Evacuation of hematoma						
(+)	1.6 (113/7283)	0.988 (0.618–1.580)	0.988	2.1 (21/979)	0.417 (0.179–0.970)	0.042
(−)	0.3 (110/43,692)	Reference		0.5 (40/8436)	Reference	
Decompressive craniectomy						
(+)	3.3 (69/2120)	2.292 (1.562–3.363)	< 0.001	4.0 (15/375)	1.468 (0.671–3.211)	0.337
(−)	0.3 (154/48,855)	Reference		0.5 (46/9040)	Reference	
Lobectomy						
(+)	3.1 (11/354)	0.909 (0.459–1.799)	0.785	1.6 (1/62)	0.547 (0.070–4.292)	0.566
(−)	0.4 (212/50,621)	Reference		0.6 60/9353	Reference	
Duraplasty						
(+)	3.2 (7/222)	1.165 (0.510–2.662)	0.717	0.0 (0/45)	NA	–
(−)	0.4 (216/50,753)	Reference		0.7 (61/9370)	Reference	
Cranioplasty						
(+)	2.1 (8/382)	1.522 (0.697–3.324)	0.292	1.8 (1/55)	0.877 (0.111–6.933)	0.901
(−)	0.4 (215/50,356)	Reference		0.6 (60/9360)	Reference	
External ventricular drainage						
(+)	3.1 (19/619)	1.584 (0.915–2.740)	0.100	5.4 (9/167)	2.790 (1.113–6.992)	0.029
(−)	0.4 (204/50,356)	Reference		0.6 (52/9248)	Reference	
Repeat surgical procedure performed within 48 h of first surgery						
Craniotomy						
(+)	4.6 (36/776)	3.044 (1.952–4.746)	< 0.001	4.6 (7/152)	4.372 (1.661–11.503)	0.003
(−)	0.4 (187/50,199)	Reference		0.6 (54/9263)	Reference	
Burr hole surgery						
(+)	4.4 (8/180)	3.387 (1.531–7.492)	0.003	7.3 (4/55)	6.368 (1.825–22.224)	0.004
(−)	0.4 (215/50,795)	Reference		0.6 (57/9360)	Reference	

*n/N* number of patients with posttraumatic meningitis/number of patients, *TBI* traumatic brain injury, *ED* emergency department, *OR* odds ratio, *CI* confidence interval, *NA* not applicable

*When assessing one variable, we adjusted other variables listed in this table

## Discussion

Using data from the JTDB as a nationwide hospital-based trauma registry in Japan, this study revealed that factors such as male sex, multiple trauma, basilar skull fracture, CSF leakage, burr hole surgery in the emergency department, burr hole surgery and craniotomy as operative methods for the first surgery, decompressive craniectomy and external ventricular drainage as procedures performed during the first neurosurgical operation, and repeat surgery within 48 h of the first surgery were associated with posttraumatic meningitis among patients with traumatic brain injury patients with head AIS score 3 or more. In the subgroup analysis, burr hole surgery in the emergency department, craniotomy, decompressive craniectomy, and repeat surgery within 48 h were associated with posttraumatic meningitis in patients with single head trauma. In contrast, in multiple trauma patients, craniotomy, external ventricular drainage, and repeat surgery within 48 h were positively associated with posttraumatic meningitis, whereas evacuation of hematoma was negatively associated with it. This study revealing the association of posttraumatic meningitis with various factors provides important clues for the prevention of posttraumatic meningitis in patients with traumatic brain injury patients with head AIS score 3 or more.

First, burr hole surgery in the emergency department was associated with posttraumatic meningitis in this study. When traumatic brain injury patients with acute epidural hematoma and/or acute subdural hematoma suffer from a rapid loss of consciousness, burr hole surgery is performed in the emergency department as an emergency procedure before damage to the brainstem becomes irreversible [23–25]. In addition, burr hole surgery is performed in the emergency department by surgeons and/or emergency physicians as an emergency procedure before patient transport from an area without neurosurgeons to a distant medical institution [10, 26]. However, the present study did not sufficiently reveal why burr hole surgery in the emergency department was associated with the occurrence of posttraumatic meningitis.

Second, decompressive craniectomy and external ventricular drainage were also associated with posttraumatic meningitis as procedures performed during the first neurosurgical operation. It was previously reported that decompressive craniectomy reduces intracranial pressure (ICP) in patients with high ICP due to traumatic brain injury [11–15, 27, 28]. However, dura and skin can sometimes not be adequately sutured due to excessive cerebral edema in patients, whose ICP is extremely high. There were also several reports on the positive association between external ventricular drainage and meningitis [8, 9, 29]. The endocranial space, which normally has no communication with the outside, is connected with the outside by decompressive craniectomy and external ventricular drainage, and bacteria might invade the endocranial space and cause meningitis. Early cranioplasty or removal of the ventricular drain could help to prevent posttraumatic meningitis in these patients [29].

Third, repeat surgery within 48 h of the first surgery have previously been associated with posttraumatic meningitis. Repeat surgery is often performed in head trauma patients because of intracranial hematoma on the opposite side after the initial surgery or cerebral edema after brain contusion. Long operation time and repeat surgery were associated with postoperative meningitis in patients undergoing neurosurgery [9]. In addition, not only trauma but also therapeutic interventions such as surgery affect the immune response of the trauma patients [30]. Therefore, the second blow caused by the neurosurgical operation may result in cerebral edema and hematoma on the opposite side after the initial surgery. Furthermore, this second blow influencing the patient’s immune response might also be associated with posttraumatic meningitis. If ICP is controlled by intensive care such as osmotic diuresis and hypothermia, decompressive craniectomy, or repeat surgery for TBI are unnecessary to control ICP. Therefore, intensive care for ICP control excluding external ventricular drainage may be helpful to prevent posttraumatic meningitis.

Finally, multiple trauma was associated with posttraumatic meningitis. Compared with single head trauma, multiple trauma is more invasive to the body and might be related to the occurrence of posttraumatic meningitis due to deterioration of the immune system in multiple trauma patients [31–34]. However, subgroup analysis showed that the factors associated with posttraumatic meningitis were different between single head injury and multiple trauma. Although the reason for this result was unclear, more invasive procedures were performed on multiple trauma patients than on single head injury patients, which could result in decreased immunity and the subsequent occurrence of posttraumatic meningitis. It is unclear why the incidence of posttraumatic meningitis was significantly lower in the trauma patients with the evacuation of hematoma, and further studies are needed. In addition, if an injury site such as that on the extremities is at risk for infection, the prevention of infection such as with the administration of antibiotics would be necessary.

### Limitations

There are some limitations in this study. First, because we extracted data on meningitis from a trauma registry, we did not obtain information on how the diagnosis of meningitis was made. However, because the morbidity rate of the patients with posttraumatic meningitis was 0.4 to 1.4% in the previous studies on posttraumatic meningitis [4, 35], and the rate in the present study was equivalent to the rates in these studies, we believe that the diagnostic accuracy would be appropriate. Second, information on the administration of antibiotics in each patient was unknown in this study, but it would be important, as the administration of antibiotics before the occurrence of posttraumatic meningitis might lead to its prevention. However, antibiotic treatments based on the Surviving Sepsis Campaign Guidelines were performed in many intensive care units in Japan [36]. Therefore, similar administration of antibiotic treatments would have been performed on the present study patients. Third, CSF leakage in patients with basilar skull fracture was assessed in this study, but CSF leakage due to frontal sinus injury could not be assessed, because there were no data on this condition. Finally, this study was an observational study, and there may be some unknown confounding factors.

## Conclusions

By use of a nationwide trauma registry in Japan, this study revealed that factors such as male sex, multiple trauma, basilar skull fracture, CSF leakage, burr hole surgery in the emergency department, burr hole surgery and craniotomy as initial operative methods, decompressive craniectomy, and external ventricular drainage, as procedures performed during the neurosurgical operation, and repeat surgery within 48 h of the first surgery were associated with posttraumatic meningitis in traumatic brain injury patients with head AIS score 3 or more. Our findings suggest that the prevention of infection such as the administration of antibiotics might be of help for traumatic head injury patients who require decompressive craniectomy or reoperation to prevent posttraumatic meningitis.

## Data Availability

The data that support the findings of this study are available from the JTDB, but the availability of these data is restricted.
